# Influence of the abstinence period on human sperm quality: analysis of 2,458 semen samples

**DOI:** 10.5935/1518-0557.20170052

**Published:** 2017

**Authors:** Vanessa A. Comar, Claudia G. Petersen, Ana L. Mauri, Mariana Mattila, Laura D. Vagnini, Adriana Renzi, Bruna Petersen, Andreia Nicoletti, Felipe Dieamant, Joao Batista A. Oliveira, Ricardo L. R. Baruffi, José G. Franco Jr.

**Affiliations:** 1 Center for Human Reproduction Prof. Franco Jr., Ribeirão Preto, SP, Brazil; 2 Paulista Center for Diagnosis Research and Training, Ribeirão Preto, SP, Brazil

**Keywords:** abstinence period, sperm parameters, DNA fragmentation, concentration, motility, volume

## Abstract

**Objective:**

The aim of this study was to evaluate the influence of different periods of abstinence on conventional semen parameters as well as functional parameters in human semen, including mitochondrial function, chromatin packing and sperm DNA fragmentation.

**Methods:**

We recruited a cohort of 2,458 men undergoing infertility investigation. Semen analyses were performed according to WHO guidelines/morphology-motile sperm organelle morphology examination/MSOME. For DNA integrity analysis, the percentages of DNA fragmentation (TUNEL), abnormal chromatin packaging/underprotamination (chromomycin A3/CMA_3_), abnormal mitochondrial membrane potential (MMP/MitoTracker Green), and apoptosis (annexin-V) were recorded. Associations between the sexual abstinence period and sperm parameters were assessed using Spearman correlation. For group comparisons, the subjects were categorized according to the sexual abstinence period (SAP) into three groups: SAP <2 days, SAP 2-5 days, and SAP >5 days.

**Results:**

The duration of abstinence had a statistically significant positive influence on sperm concentration and volume, the number of leukocytes and a statistically significant negative influence on sperm motility and vitality. The percentages of DNA fragmentation and MMP (mitochondrial damage) worsened with the increased duration of abstinence. The percentage of sperm protamination was statistically significantly increased with abstinence.

**Conclusion:**

Increase in the sexual abstinence period influences sperm quality. This study reinforces the importance of the duration of ejaculatory abstinence on semen parameter variation. It highlights the deleterious effect of increased abstinence on DNA damage, which is most likely associated with ROS (mitochondrial damage/number of leukocytes). The increase in chromatin packaging can represent a protective feature for DNA.

## INTRODUCTION

Semen analysis implies the evaluation of several characteristics of the ejaculate with the intent of estimating the reproductive chance/probability of an individual. Studies have found that human semen samples vary over time and this may be due to three principal factors: (i) pre-analytical influences (in the case of semen: sexual abstinence period and transport of the sample to the laboratory); (ii) analytical randomization (precision) and systematic error (bias); and (iii) inherent biological variation (WHO, 2010; Castilla *et al.*, 2006; Alvarez *et al.*, 2003; Cardona Maya *et al.,* 2007). The search for predictors of male fertility has resulted in the standardization of procedures for the evaluation of human semen; an example of this is the set of guidelines developed by the WHO, to process seminal samples based on global demographic population studies (WHO, 2010). This effort has made a solid contribution to semen analysis and has provided a better understanding of human sperm quality (Mayorga-Torres *et al.*, 2015).

Human sperm is produced in the seminiferous tubules, and then stored in the epididymis for future release (Sullivan *et al.*, 2016; Mayorga-Torres *et al.,* 2015). Unlike other species, for the male mammalian gamete to mature and acquire fertilization potential it must pass through the epididymis, where it undergoes a series of physiological and biochemical changes (Sullivan *et al.*, 2016). Epididymal transit time was estimated between 2 to 11 days (Mayorga-Torres *et al.,* 2015). The variation is influenced by ejaculatory frequency (Mayorga-Torres *et al.,* 2015). The World Health Organization (WHO) recommends a period of abstinence 2-7 days prior to collection for standard semen evaluation (WHO, 2010). However, recent studies have suggested that shorter abstinence is better for assisted reproductive techniques (ART) than the abstinence recommended by the WHO for performing seminal diagnostic analysis. The abstinence period is important to ensure both the quantity and the quality of spermatozoa, required for successful, natural and assisted conception (Lehavi *et al.*, 2014).

Long-term abstinence leads to the buildup of spermatozoa in the epididymis, and it may increase their exposure to the harmful effects of reactive oxygen and nitrogen species (ROS and RNS) generated mainly by granulocytes during maturation and storage in the epididymis. Thus, spermatozoa are susceptible to oxidative attack, and this has been correlated with decreased sperm motility, lipid peroxidation, DNA damage and compromised fertilization rates (Agarwal & Said, 2003; Du Plessis *et al.*, 2010). Standard semen analysis does not identify sperm senescence or functional impairment. Examining the effect of abstinence at the functional level requires more sensitive sperm tests such as DNA fragmentation (Amann *et al.*, 2009). However, there is a lack of consensus on the exact influence of the abstinence period on both conventional and functional parameters. Thus, the impact of sexual abstinence on conventional sperm parameters is still debatable (Levitas *et al.*, 2005; Gosálvez *et al.*, 2011; Sánchez-Martín *et al.*, 2013; Agarwal *et al.*, 2016; Mayorga-Torres *et al.*, 2015; 2016).

Based on the above evidence, the objective of this study was to evaluate the influence of different periods of abstinence on conventional seminal parameters, as well as functional parameters in human semen, including mitochondrial function, chromatin packing and sperm DNA fragmentation.

## MATERIALS AND METHODS

### Population

We recruited a cohort of 2,458 men undergoing infertility investigation. In the first evaluation, each man was asked to deliver a semen sample, and the abstinence period was recorded. No instruction about the abstinence period was given before semen collection for the study. Exclusion criteria were azospermia, any known reproductive tract pathology in the last six months, any hormonal therapy in the past six months and chronic medical disorders. Written informed consent was obtained from all participants, and this study was approved by the institutional review board of local ethics committee.

### Sample collection

Semen samples were collected in sterile containers by masturbation. One portion of each semen sample was used for analysis according to the WHO guidelines (WHO, 2010). Another portion of each semen sample was immediately processed for morphological analysis by motile sperm organelle morphology examination (MSOME). The remainder of the semen samples was immediately processed for sperm DNA fragmentation analysis using the TdT (terminal deoxyribonucleotidyl transferase)-mediated dUTP nick-end labelling (TUNEL) assay; sperm apoptosis analysis was carried out using the Annexin V assay, sperm chromatin packing/underprotamination using Chromomycin A3 (CMA_3_) staining, and sperm mitochondrial membrane potential/mitochondrial damage using MitoTracker Green.

### Determination of morphology by MSOME

MSOME procedures were performed as described previously (Silva *et al.*, 2012; Oliveira *et al.*, 2014). At least 200 motile spermatozoa per sample were evaluated, and the percentages of normal spermatozoa were determined.

### Determination of sperm DNA fragmentation

DNA fragmentation in spermatozoa was measured using the TdT (terminal deoxyribonucleotidyl transferase)-mediated dUTP nick-end labelling (TUNEL) assay, which was performed using an *in-situ* cell death detection kit, with tetramethylrhodamine-labelled dUTP (Roche, Monza, Italy), as described previously (Vagnini *et al.*, 2007; Oliveira *et al*., 2014). The final evaluation was achieved using a fluorescent (Olympus BX 50) microscope and the percentage of TUNEL-positive spermatozoa was determined. At least 200 sperm were evaluated on each slide, with the appropriate filter.

### Determination of sperm chromatin packaging/underprotamination

Sperm protamine deficiency (underprotamination/chromatin) packaging was measured using chromomycin A_3_ (CMA_3_), as described previously (Franco *et al*., 2012). The percentage of positive spermatozoa was determined by direct observation in four fields, using a fluorescent microscope (Olympus BX 50) and the percentages of spermatozoa with abnormal chromatin packaging were determined. At least 200 sperm were evaluated on each slide, with the appropriate filter.

### Determination of sperm apoptosis

Sperm apoptosis was measured using annexin-V, a calcium-dependent phospholipid-binding protein with a high affinity for phosphatidylserine that is present on the inner leaflet of the sperm membrane, except for apoptotic sperm, in which phosphatidylserine is externalized. The sperm suspensions (1x10^6^cells/ml) were incubated with 1 µL annexin-V, 1 µL propidium iodide (Dead Cell Apoptosis Kit with Annexin V Alexa Fluor^®^ 488 & Propidium Iodide (PI); Molecular Probes, Eugene, OR) and 1µL Hoechst 33342 at room temperature for 15 min in a dark environment. PI is a dye impermeable to live cells. After incubation, the suspension was centrifuged at 800*g* for 10 min, and the pellet was mounted on poly-l-lysine-coated slides for examination under a fluorescent microscope (Olympus BX 50). At the analysis, subpopulations of sperm could be identified: annexin V(−)/PI(−) - live intact sperm; annexin V(+)/PI(−) - early apoptotic cells; annexin V(+/−)/PI(+) - necrotic cells. The percentages of apoptotic cells (defined number of spermatozoa that were stained in green and positive for annexin-V but excluded the positive for red propidium iodide dye, divided by the total number of spermatozoa) were determined. At least 200 sperm were evaluated on each slide, with the appropriate filter.

### Determination of sperm Mitochondrial membrane potential

Sperm mitochondrial membrane potential (MMP), an indicator of sperm functionality, since it expresses sperm mitochondrial function, was determined using MitoTracker Green (Molecular Probes, Eugene, OR, USA). The live sperm suspensions were incubated in PBS containing 20 nM MitoTracker for 20 min at 37°C. To stain the sperm DNA, the samples were subsequently incubated with Hoechst 33342 (10min, 37°C). After incubation, the suspension was centrifuged at 800g (10 min), and the pellet was mounted on a microscope slide. The green fluorescence in the midpiece determined the active mitochondria. Sperm samples were examined using a fluorescent microscope (Olympus BX 50) equipped with a triple band pass filter, and the percentages of spermatozoa with altered MMP/mitochondrial damage (i.e., absence of green fluorescence) were determined. At least 200 sperm were evaluated on each slide, with the appropriate filter.

### Quality control

To control for intra-observer and inter-observer variability, multiple fractions of semen samples were obtained from randomly selected patients. Each sample was studied at least three times by the same examiner (blinded for subject identity). An intra-observer and an inter-observer variation of ≈ 0.5-1% and 0.5-7%, respectively, were obtained for each parameter analyzed: semen parameters according to the WHO guidelines, normality of the spermatozoon, normality of nuclear form, normality of chromatin, spermatozoa with any nuclear vacuoles, spermatozoa with vacuoles occupying >50% of the nuclear area, sperm TUNEL-positive, sperm CMA_3_-positive, sperm Annexin V-positive, and sperm MitoTracker Green-positive. These variations are comparable to those of classical sperm quality parameters (Auger *et al.,* 2000).

### Sample size

The sample size was calculated by making a comparison between two proportions. A sample size of 200 sujects in each group has 80% power to detect an increase of 15%, with a significance level of 0.05 (two-tailed).

### Statistical analysis

The data was analyzed using the StatsDirect statistical software (Cheshire, UK). Potential confounders (age, body mass index (BMI), smoking, alcohol, varicocele and vitamin use) were also noted. Regression and correlation analyses with continuous variables (age, BMI, sperm volume, sperm pH, sperm concentration, percentage of spermatozoa with progressive motility, percentage of total sperm motility, percentages of normal spermatozoa, number of leucocytes and percentage of live spermatozoa (vitality)) were performed using the Spearman rank correlation test. For dichotomous variables (smoking, alcohol, varicocele and vitamin use) correlations were performed using logistic regression.

For group comparisons, the following sexual abstinence period (SAP) were used as cut-off points to divide the subjects into three groups: SAP< 2 days, SAP 2-5 days and SAP >5 days. Mann-Whitney U test, Student's t-test and the chi-squared test were used as appropriate. The level of significance was set at *p*<0.05.

## RESULTS

### General population characteristics

The regression analysis did not show a correlation between days of abstinence and men's age, history of fathering at least one child (or generating a pregnancy that had ended in miscarriage), time of infertility, tobacco use, regular alcohol use, presence of varicocele and vitamin supplement use. The comparison between the three SAP Groups confirmed these results. Table 1 summarizes the data.

**Table 1:** Correlation between general population characteristics and Sexual Abstinence Period.

Characteristic	Regression analysis			Sexual Abstinence Period Groups				
	r*/OR**	95% Confidence Interval	*p*	Total n:2458	<2 days n:244 (10%)	2-5 days n:1932 (78.6%)	>5 days n:282 (11.4%)	*p*
Age (years)	r: 0.04	-0.002 to 0.08	0.06	37.7±6.5	37.6±6.3	37.6±6.4	38.3±7.1	0.21
BMI	r:0.01	-0.03 to 0.06	0.53	28.6±4.4	28.8±4.3	28.6±4.4	28.4±4.4	0.77
Fathered at least one child	OR: 1.00	0.98 to 1.02	0.61	31.7% (779/2458)	32.4% (79/244)	32.2% (622/1932)	27.7% (78/282)	0.30
Time of infertility (years)	r: 0.03	-0.01 to 0.07	0.13	4.0±3.3	3.8±2.2	4.0±2.1	4.2±3.2	0.21
Tobacco use (%)	OR: 0.98	0.93 to 1.03	0.44	11.4% (281/2458)	10.2% (25/244)	11.7% (226/1932)	10.6% (30/282)	0.77
Regular alcohol use (%)	OR: 0.99	0.97 to 1.01	0.57	66.1% (1624/2458)	64.3% (157/244)	66.6% (1287/1932)	63.8% (180/282)	0.53
Varicocele (%)	OR: 1.01	0.99 to 1.03	0.17	15.9% (391/2458)	14.9% (36/244)	16.3% (315/1932)	14.2% (40/282)	0.60
Vitamin supplement use (%)	OR: 0.98	0.95 to 1.03	0.56	16.2% (397/2458)	17.2% (42/244)	16.2% (313/1932)	14.9% (42/282)	0.77

* Spearman's correlation.

** Logistic regression.

r: Spearman's rank correlation coefficient.

OD: odds ratio.

Values within rows with the same superscript letter were significantly different.

### Semen quality/General semen parameters

The regression analysis did not show a correlation between sexual abstinence, sperm pH and morphology (*p*>0.05). However, significant (*p*<0.05) decreases in progressive motility, total sperm motility and sperm vitality with increasing periods of sexual abstinence were noticed. On contrary, the duration of abstinence had a statistically significant positive influence on sperm concentration (*p*<0.05). The group comparison reinforces these outcomes. Table 2 summarizes the data.

**Table 2:** Correlation between general semen parameters and sexual abstinence period.

Semen Parameters	Regression analysis			Sexual Abstinence Period Groups				
	Spearman's rank correlation	95% Confidence Interval	*p*	Total n:2458	<2 days n:244	2-5 days n:1932	>5 days n:282	*p*
pH	r: -0.01	-0.05 to 0.02	0.52	8.0±0.4	8.0±0.6	8.0±0.5	8.0±0.4	0.28
Volume (ml)	r: 0.10	0.08 to 0.16	0.0004	2.7±1.5	2.3±1.2^a,b^	2.7±1.6^a^	2.8±1.7^b^	^a^0.0001 ^b^0.0004
Concentration (mlx10^6^)	r: 0.12	0.088 to 0.16	<0.0001	71.9±61.1	60.7±50.0^a,b^	70.4±61.1^a,c^	92.1±65.8^b,c^	^a^0.02 ^b,c^<0.0001
Progressive motility (rapid + slow progression) (%)	r: -0.10	-0.10 to -0.02	0.0007	56.1±16.7	58.3±16.2^a,b^	56.1±16.9^a,c^	53.8±16.1^b,c^	^a^0.03 ^b^0.0004 ^c^0.01
Total motility (%)	r: -0.10	-0.13 to -0.05	<0.0001	63.0±16.2	65.5±15.6^a,b^	63.1±16.2^a,c^	59.7±16.2^b,c^	^a^0.01 ^b^<0.0001 ^c^0.0002
Normal sperm forms (%)	r: 0.03	-0.008 to 0.07	0.12	0.8±1.4	0.7±1.0	0.9±1.4	1.0±1.8	0.77
Leukocytes (x10^6^/ml)	r: 0.10	0.03 to 0.11	0.0001	0.4±0.9	0.3±0.6^a,b^	0.4±0.9^a^	0.5±1.2^b^	^a^0.002, ^b^0.0002
Vitality (%)	r: -0.10	-0.13 to -0.05	0.006	64.5±14.9	66.9±14.0^a,b^	65.0±15.0^a,c^	61.3±14.8^b,c^	^a^0.03 ^b,c^<0.0001

Values within rows with the same superscript letter were significantly different.

### Sperm DNA fragmentation/sperm chromatin packing/sperm apoptosis/sperm mitochondrial membrane potential

We found a positively significant correlation between sexual abstinence duration and sperm DNA fragmentation (percentage of sperm TUNEL positive) and percentage of sperm abnormal mitochondrial membrane potential/mitochondrial damage (*p*<0.05). However, there was a significant decrease in the percentage of sperm CMA_3_ positive with increasing sexual abstinence period (*p*<0.05). There was no correlation (*p*>0.05) between abstinence and sperm apoptosis (percentage of sperm annexin V positive). The results of the group comparisons confirm the regression analysis results. Table 3 summarizes the results.

**Table 3:** Correlation between sperm DNA fragmentation, sperm chromatin packing, sperm apoptosis and sperm mitochondrial membrane potential according to the sexual abstinence period.

Semen Parameters	Regression analysis			Sexual Abstinence Period Groups				
	Spearman's rank correlation	95% Confidence Interval	*p*	Total n:2458	<2 days n:244	2-5 days n:1932	>5 days n:282	*p*
DNA fragmentation (%)	r: 0.12	0.16 to 0.44	<0.0001	15.4±8.5	14.5±8.2^a^	15.3±8.4^b^	17.1±9.0^a,b^	^a^0.001 ^b^0.002
Abnormal MMP (%)	r: 0.10	0.03 to 0.16	0.003	25.7±16.4	23.3±14.0^a^	25.6±16.5	28.6±17.5^a^	^a^0.01
CMA_3_ positivity (%)	r: -0.12	-0.14 to -0.04	<0.0001	56.1±15.2	59.8±15.3^a,b^	56.1±15.2^a,c^	53.2±14.2^b,c^	^a^0.005 ^b^0.0002 ^c^0.02
Apoptosis (%)	r: 0.01	-0.03 to 0.06	0.21	19.2 ±7.9	19.3±8.5	19.1±7.9	20.2±7.3	0.17

Values within rows with the same superscript letter were significantly different.

## DISCUSSION

The effects of sperm abstinence period on semen quality have been discussed in the literature, but the results are not consistent. We observed in our results that the main changes in different abstinence periods were in semen volume, semen concentration, progressive motility, leukocyte concentration, sperm vitality, DNA fragmentation percentage, CMA_3_ positivity percentage and abnormal MMP percentage. We did not find a major change in any of the other sperm parameters available, such as semen pH, morphology and apoptosis percentage.

Our results found that an increased abstinence period is associated with decreases in sperm vitality, sperm progressive motility and CMA_3_ positivity. Several studies have reported similar results, noting an inverse correlation between sperm motility (Rao *et al.*, 2015; Lehavi *et al.*, 2014; Makkar *et al.*, 2001; Hornstein *et al.*, 1992; Tur-Kaspa *et al.*, 1994) and sperm vitality (Mayorga-Torres *et al.*, 2016; Levitas *et al.*, 2005; Agarwal *et al.*, 2016) with semen abstinence period. There is no consensus in the literature, and another author did not observe any correlation between abstinence duration and some of these semen parameters (Mayorga-Torres *et al.*, 2015).

The semen volume, in this study, was significantly increased after longer abstinence (in days). The findings regarding semen volume are compatible with data reported in similarly controlled studies (Rao *et al.*, 2015, Lehavi *et al.*, 2014; Sauer *et al.*, 1988; Blackwell *et al.*, 1992) and with population-based studies (Matilsky *et al.*, 1993; Padova *et al.*, 1998; Le Lannou *et al.*, 1986; Schwartz *et al.*, 1979; Jouannet *et al.*, 1981; Mortimer *et al.*, 1982; De Jonge *et al.*, 2004). 

Regarding sperm motility, there was a decrease with longer periods of abstinence. The findings of this study is in accordance with previously published studies (Rao *et al.*, 2015; Lehavi *et al.*, 2014, Makkar et al., 2001; Hornstein *et al.*, 1992; Tur-Kaspa *et al.*, 1994) that noted that lower abstinence time improves the percentage of sperm motility, and particularly the total motile sperm counts among patients with male factor infertility. Therefore, pooling sequential ejaculates was recommended to increase success rates (Tur-Kaspa *et al.*, 1994). One of the reasons for the relatively fast reduction in this parameter can be related to the increased production of ROS. Oxidative stress is a condition associated with an increased rate of cellular damage induced by oxygen and oxygen-derived oxidants, known as reactive oxygen species (Sikka *et al.*, 1995). Damaged spermatozoa (Iwasaki *et al.*, 1992) or infiltrating leukocytes (Kessopoulu *et al.*, 1994) are source of ROS, which is correlated with decreased sperm motility. In fact, higher levels of ROS have been found in semen of infertile men, when compared with the semen from fertile patients (Levitas *et al.*, 2005; Iwasaki *et al.*, 1992, Aitken *et al.*, 1991).

To summarize, our results show that semen variables such as semen volume and total sperm count, increase with semen abstinence period. Conversely, the longer the SAP, the greater the semen volume, sperm concentration, leucocyte concentration, DNA fragmentation and abnormal mitochondrial membrane potential.

Further, the sperm concentration in larger abstinence periods was substantially higher, and this can be ascribed to the fact that reserves were available and stored in the epididymis. Due to the lower abstinence period, the reserves were depleted and total sperm count decreased with shorter abstinence periods (Rao *et al.*, 2015). In our study, the sperm concentration was significantly increased after both 2-5 and >5 days' abstinence in comparison with a 2 day-abstinence. These findings raise a question concerning daily sperm production. We have found that 2-5 and >5 days' abstinence produced the greatest semen volumes and sperm concentrations in comparison with 2 day's abstinence.

Similarly to its influence on sperm motility, an increase in semen oxidative stress related to the sperm DNA fragmentation index was found in patients with male factor infertility, compared with cases of fertile donors (Levitas *et al.*, 2005; Saleh *et al.*, 2003). Some researchers have reported a relationship between oxidative stress and male infertility, and suggest that treatments for infertility in these men should include strategies to reduce oxidative stress (Pasqualotto *et al.,* 2000).

DNA integrity of spermatozoa has been considered an important parameter in fertility studies (Mayorga-Torres *et al.*, 2013; Evenson 2013; Evenson *et al.*, 1999; Gil-Villa *et al.*, 2009; 2010; Rodríguez *et al.*, 2011). Evenson *et al.* found that semen samples with a DNA fragmentation index of more than 29% has an increased likelihood of reduced fertility (Mayorga-Torres *et al.*, 2015; Evenson *et al.*, 1999).

The results from the present study showed that the DNA fragmentation percentage increased in the longer periods of abstinence. Similar studies have shown that shorter abstinence periods lead to a greater reduction in the incidence of sperm DNA fragmentation and an increase on pregnancy rates after assisted reproductive techniques (Sánchez-Martín *et al.*, 2013; Gosálvez *et al.*, 2011). Variations in the results reported in the literature may be related to the technique used and/or the population analyzed.

The quality of sperm chromatin packaging has been reported to play an important role during fertilization and early embryo development. Several investigators have used chromomycin A3 to evaluate sperm chromatin packaging in relationship to IVF outcome, similar to the present study. chromomycin A3 values have been reported to be inversely correlated with sperm count and *in vitro* fertilization (Lolis *et al*., 1996; Iranpour *et al.*, 2000). In contrast to DNA fragmentation data, there appeared to be a negative influence of a short abstinence interval (24 hours) on sperm chromatin packaging. We found that the percentage of sperm with immature chromatin was significantly higher after 2 days' abstinence in comparison with after 2-5 days' abstinence and >5 days abstinence. Sperm nuclear condensation serves, in part, as a protection mechanism for the DNA. Poorly condensed chromatin is more vulnerable to the potentially deleterious influences of environmental factors, such as peroxidation.

In our study, the abnormal MMP was signicantly higher in patients who maintained a longer period of abstinence than those who maintained a shorter abstinence period. It has been well described in studies of ROS production and sperm physiology that these highly reactive chemical species play a major role in many sperm processes such as maturation, motility and capacitation (Kothari *et al.*, 2010). Nevertheless, ROS levels must be controlled within physiological boundaries as overproduction or lack of sufficient antioxidant systems can lead to the development of oxidative stress, and it may affect mitochondrial function.

In conclusion, increase in the sexual abstinence period influences sperm quality. This study reinforces the importance of the duration of ejaculatory abstinence on semen parameter variation. It highlights the deleterious effect of increased abstinence on DNA damage, which is most likely associated with ROS (mitochondrial damage/number of leukocytes). The increase in chromatin packaging can represent a protective feature for DNA. However, pitfalls of the chromomycin A3 method cannot be excluded.
